# Information Hazards in Biotechnology

**DOI:** 10.1111/risa.13235

**Published:** 2018-11-12

**Authors:** Gregory Lewis, Piers Millett, Anders Sandberg, Andrew Snyder‐Beattie, Gigi Gronvall

**Affiliations:** ^1^ Future of Humanity Institute Oxford University Oxford UK; ^2^ Centre for Health Security Johns Hopkins Bloomberg School of Public Health Baltimore MD USA

**Keywords:** Biotechnology, dual‐use research of concern, information hazards

## Abstract

With the advance of biotechnology, biological information, rather than biological materials, is increasingly the object of principal security concern. We argue that both in theory and in practice, existing security approaches in biology are poorly suited to manage hazardous biological information, and use the cases of Mousepox, H5N1 gain of function, and Botulinum toxin H to highlight these ongoing challenges. We suggest that mitigation of these hazards can be improved if one can: (1) anticipate hazard potential before scientific work is performed; (2) consider how much the new information would likely help both good and bad actors; and (3) aim to disclose information in the manner that maximally disadvantages bad actors versus good ones.

## 1. INTRODUCTION

It has long been recognized that there are biosafety risks that arise from particular biological materials, from biological toxins in a laboratory, to potentially epidemic disease, to introducing invasive species into an ecosystem. It is also widely acknowledged that the rapid and ongoing progress of biotechnology (including synthetic biology and other emerging technologies) will enhance and change this risk landscape (Gronvall, 2016; National Research Council, 2004a).

One such change is that the biological *information*, rather than the corresponding biological *materials*, is increasingly the object of greatest security concern. These are *information hazards*, defined as “a risk that arises from the dissemination of (true) information that may cause harm or enable some agent to cause harm” (Bostrom, 2011). Incidence of these risks will likely increase over time, yet our understanding of this hazard lags behind.

This article is an initial attempt to catch up. We sketch general considerations about biological information hazards, describe cases in biosecurity through this lens, and suggest approaches for managing these hazards. We hope to raise awareness of this issue among both practitioners in the biological sciences and those concerned with their misuse, and point to areas for action that may reduce risk.

## 2. AGAINST THE OPENNESS/SECRECY AXIS

A common frame to discuss hazardous information, whatever the context, is to range the competing benefits of openness and secrecy against one another. On one side are the benefits of faster scientific progress and freedom of information; on the other, the benefit of safeguarding others from harm (Casadevall et al., 2013).

Balancing these considerations in particular cases can be fraught. Many nations make the presumption that scientific research should not be restricted or held to national classification regimes. For example, in 1985, U.S. government concluded that “to the maximum extent possible, the products of fundamental research remain unrestricted” (Reagan, 1985). Germany enshrines freedom of research in its Basic Law (Art. 5/3) (Germany, 1949). Managing scientific information hazardous enough to warrant an exception to this principle is left to security classification (with “sensitive but unclassified” often used in the United States), or export control (attempted by the Dutch government for the gain‐of‐function influenza work). The use of either in the biological sciences has been criticized as inappropriate (National Research Council, 2007; Shaw, 2016)

These challenges reflect imperfect understanding of how biological information can pose a hazard. Biological information cannot be neatly segregated into the safe and open, and the hazardous and secret: much is to a greater or lesser degree “dual use”; it is also often incremental, building upon prior information that is openly available. Further, the appropriate degree of openness or secrecy is not solely intrinsic to the information in question, but also depends on the characteristics of potential good or bad actors that might (mis)use it. Given the possibility of deliberate misuse by intelligent adversaries, both openness and secrecy can backfire in surprising ways.

### 2.1. Biological Information Is Often Dual Use

Biological understanding, as well as biological technology, can be dual use. Information on potentially pandemic pathogens can be used not only by health security professionals to develop countermeasures, but also by those seeking to use disease as a weapon. Making new biotechnology easier to access can enhance the work of responsible scientists, yet also pose danger if deployed by the reckless.

Dual‐use information is widely prevalent in biology. Apparently benign discoveries (e.g., the mechanism of action of a common therapeutic agent) could potentially aid a malicious actor (e.g., by adapting the pathogen to no longer be susceptible to available therapeutic drugs). Apparently dangerous information may ultimately prove beneficial if it “raises the alarm” and informs mitigation efforts. There are not “bright lines” that circumscribe a blacklist of biological information that could only be used for harm (and thus kept secret) versus all other benign information that can be freely disseminated.

It is similarly difficult to draw clear lines around the sort of scientific work that *could* produce hazardous information. As an example, U.S. government policies on dual‐use research (U.S. Government, 2014) apply to work that both constitutes one of the seven experiments of concern (cf. National Research Council, 2004b) and is performed on a subset of organisms on the federal Select Agent List (cf. The Federal Select Agent Program, n.d.). While it is important to have clear lines drawn around regulated activities, it is nonetheless easy to imagine work that would not “fall under” this policy but would produce potentially hazardous information.

Instead, there may be a fuzzy penumbra comprising information of an intermediate degree of hazard that warrants nuanced decision making and response (National Academies of Sciences, 2017a).

### 2.2. Producers and (Mis)users of Biological Information Hazards

The degree of hazard from a given piece of biological information partly depends on the population of actors that might use it for good or ill. The people who (mis)use biological information hazards may differ from those who produce them. Anticipated characteristics of producers, users, and misusers alter the nature and degree of hazard, and thus alter how it is best mitigated.

Potential misusers may vary in their ability to translate an information hazard into a risk. Even with perfect knowledge, nuclear weaponry is only feasible for large state‐like actors, whereas a cyber vulnerability in a banking program could be exploited by a single individual. Similarly, biological information hazards imply varying constraints in degree and in kind (e.g., money, time, equipment, and tacit knowledge) on which actors have opportunity to misuse them. The same information can be hazardous if known by a rogue state, yet harmless if known by a potential bioterrorist.

Bad actors also vary in their capacity to generate hazardous information themselves. In cases where one expects a few highly sophisticated bad actors, easy‐to‐discover information hazards can be shared widely: good actors may benefit, and bad actors likely already know or are likely to rediscover the information hazard themselves. When large numbers of unsophisticated bad actors are anticipated, publicizing even easy‐to‐rediscover information hazards can be risky, as this insight forms a hurdle that many of them would be unable to clear on their own.

Actors without bad intent can generate information hazards. Similar principles may generally apply: the more powerful and sophisticated the actor, the greater its ability to generate information, and so greater the likelihood of generating information hazards.

The constraints on generating an information hazard may differ from the constraints on misusing it: an actor without the ability (or desire) to misuse an information hazard may nonetheless generate it. Those who may generate information hazards may act under different incentives from those trying to actualize or mitigate their risk. On the one hand, biological sciences tend to promote and reward sharing information: funders of life science research increasingly require data and information generated to be placed in the public domain, and tenure is seldom granted for papers the authors refrain from publishing. Conversely, funders also have policies on dual‐use research that encourage recipients to take information hazards into account. Yet, there remain few procedures, tools, or community drivers for addressing information hazards.

### 2.3. Openness of Benign Information and Secrecy of Hazardous Information Can Cause Harm

Both openness and secrecy can backfire. Information hazards generally offer a case of “openness” backfiring: information that may be intended for worthwhile use could be misused unexpectedly. Even information that seems transparently benign may have hazardous second‐order effects. The public repudiation of biological weaponry in the Geneva protocols informed Japan that Western powers thought biological weapons had utility, and inspired its own development and use of biological weapons in World War II (Carus, 2017); similarly, U.S. concern over bioterrorism prompted Al‐Qaeda to commence its own bioterror program (Wright, 2002).

Inappropriate secrecy can impair emergency response (Chernov & Sornette, 2016); it may also impair preventative efforts. Keeping others “in the dark” increases the risk of accidental misuse by well‐intentioned people. It also degrades the capacity of good actors to respond to risks posed by bad ones. Perhaps the free flow of scientific and technological know‐how is essential to understand which prospects of misuse are actually dangerous, speedily mitigating those that are, and that censorship merely acts to drive concerning research “underground” (Carlson, 2003). Wide disclosure of biological information hazards might be necessary to manage them.

Attempts to make potentially hazardous information secret at a late stage can prove counterproductive. Widely publicized opprobrium about whether information should have been released also publicizes the hazardous information in question, informs bad actors whose approaches are thought particularly dangerous, and produces perverse incentives for those seeking notoriety (or citations). “Security by obscurity” may be unreliable, but its opposite may be even worse.

## 3. CASE STUDIES OF BIOLOGICAL INFORMATION HAZARDS

The complexity of the decision making involved in the management of biological information hazards is best demonstrated through an examination of case studies. We describe several real‐world examples of legitimate biological research that presented perceived or actual information hazard risks, and that illuminate the challenges in dealing with these risks.

### 3.1. Mousepox

In 2001, a group of Australian researchers working on developing a population control measure for mice (Jackson & Ramshaw, 2010) published a paper that raised biosecurity concerns. They added IL‐4, an immunomodulatory gene, into the viral genome of ectromelia (mousepox), and found that the modified mousepox killed mice vaccinated for the unmodified mousepox. The mousepox virus is related to smallpox, an eradicated orthopoxvirus that killed more people in the 20th century than all wars combined. The biosecurity concern was that if the same immunomodulatory gene was incorporated into deliberately reintroduced smallpox, it may also escape the vaccine.

Publication prompted debate on whether the information in the paper was worth publishing from a standpoint of security, responsibility, and safety. The Australian government rested much of its decision that publication was safer on the thought: What if another group decided to add an immunomodulatory gene to a contagious virus, and not consider the possibility that they might create a much more dangerous virus? (Nowak, 2001). Others thought that information about how smallpox virus might be manipulated, a virus that kills 30% of those it infects, only presented a danger to society, and should not have been published (Preston, 2002). Stories circulated about various nonpublished events where researchers threw out their virus stocks after a surprising and potentially hazardous result. Another Australian research group from the same institution thought that the mousepox work could have been predicted, and thus should not have been attempted in the first place (Müllbacher & Lobigs, 2001). Finally, some experts thought that the paper did the security field a service, as it demonstrated vaccine alone would be insufficient, and prompted efforts to develop drugs that could counteract smallpox infection (Chen et al., 2011).

### 3.2. Gain‐of‐Function Influenza

The gain‐of‐function influenza controversy started in September 2011 (Gronvall, 2013). Ron Fouchier, a virologist at the Erasmus Medical Center in the Netherlands, presented work at a conference in Malta about how they had pushed the H5N1 avian influenza virus—a virus that was quite lethal in humans but that had not yet evolved to become reliably transmissible from person to person—to become contagious in a mammal model in his laboratory. The mutations in the modified virus were all found in natural avian influenza strains, though not all in the same strain, which could suggest that the virus was already on its way to evolving properties of transmission. The Fouchier paper was submitted to *Science*. Another prominent virologist, Yoshihiro Kawaoka, who has laboratories at the University of Wisconsin–Madison and University of Tokyo, performed a different type of experiment that produced the same general conclusion that H5N1 could be transmissible among humans. The work was funded by the U.S. government.

At that time, the submitted papers found their way into the consideration of the National Science Advisory Board for Biosecurity (NSABB), an advisory body to the U.S. government. The committee recommended that the papers not be published. The path to consider information hazards proceeded from that point. A moratorium on influenza research of this type was declared. Many high‐level government meetings took place involving the United States and the Netherlands, but also the World Health Organization (WHO), professional societies, and the influenza research community. In the end, the moratorium was mostly lifted for gain‐of‐function research, with some new policies put into place to vet proposed research prior to funding.

The arguments for or against the publication of the research, however, have largely been unresolved. Those who were against the research and publication thought that the scientists should not have been doing the work in the first place, and that the scientists were creating rather than addressing known risks. Opponents of the research worried that once the sequences of the modified viruses were published, it would be significantly less challenging for a malicious actor to create a damaging influenza strain. The other side of this argument maintained that the knowledge uncovered by the research was important for surveillance of avian influenza strains. Since 2003, the WHO reports that there have been 860 laboratory‐confirmed cases of H5N1, of which 454 resulted in death, a case fatality rate of 58% (The World Health Organization, n.d.). Considering that the most devastating influenza pandemic of the 20th century, the 1918 influenza pandemic, killed at least 50M people had “only” a 2.5% fatality rate (Taubenberger & Morens, 2006), H5N1 could present a much greater risk to public health. Knowing which genetic mutations to look for would be important for surveillance.

### 3.3. Botulinum Toxin H

Another concrete example of a biosecurity‐relevant information hazard came in 2013 with the identification of a novel subtype of botulinum toxin. Botulinum toxin regularly appears on lists of agents of concern, for example, being on both the export control list of the Australia Group (The Australia Group, 2017) and the U.S. Select Agent List (The Federal Select Agent Program, n.d.). Two papers argued that the newly discovered neurotoxin was encoded in a sequence that differed “substantially from the sequences of the 7 known… toxin types A–G” (Dover, Barash, Hill, Xie, & Arnon, 2014), and was not neutralized by the antitoxins that are effective against toxin types A–G (Barash & Arnon, 2014). Dual‐use concerns were cited in the rationale published alongside the articles (Hooper & Hirsch, 2014). The authors of the papers self‐censored, actively seeking permission to publish their findings without depositing the sequence encoding the new toxin into a public repository: access to the sequence data would potentially allow a bad actor to acquire this specific agent more easily than through other routes.

At first glance, this is an example of scientists attempting to act responsibly and reduce the overall biorisk posed by botulinum toxin by avoiding an information hazard. A secondary impact of this decision, however, may have increased risk. The journal accepted the proposal not to publish the sequence “[b]ecause no antitoxins as yet have been developed to counteract the novel *C. botulinum* toxin.” By restricting access to this toxin and the sequence used to create it, the number of research groups able to work on developing appropriate medical countermeasures was also severely restricted. This, in turn, would likely have impacted ability to develop a treatment. When strains of the toxin‐producing organism were shared with other labs, their assessment of both the difference in sequence and the efficacy of antitoxins contradicted the earlier findings (Maslanka et al., 2016). This suggests that being overcautious with information hazards can also complicate effective risk assessment.

This is also an excellent example of the complexity of trading off secrecy and openness. As a commentary to the reassessment of the later paper noted:

Although it is ethical to identify and mitigate DURC, it is also an ethical imperative to enable others to counter potential harm with good. With critical national security and public health at stake, it is unethical to impede research competitors for personal, professional, or commercial motives. Likewise, excessive government regulation is not helpful if it slows the progress of countermeasure development (Keim, 2016, p. 333).

## 4. PRINCIPLES TO MANAGE BIOLOGICAL INFORMATION HAZARDS

The examples above illustrate the challenges of dealing with candidate biological information hazards. We offer below some suggestions that may aid the management of similar situations in future.

### 4.1. Predict Which Work Is Most Likely to Generate Information Hazards

In many instances, the emergence of an information hazard could not have been predicted. Yet, in other cases (perhaps including those we list above), this risk could have been anticipated, and decision making about how to best manage this risk better performed “in advance” rather than after the hazardous information has been generated, or when the results are about to be published.

We do not believe that “risky” bioscience can be clearly demarcated. Although select agents or experiments of concerns can be heuristics for enhanced caution, we urge stakeholders to be mindful of the prospect for misuse of work that does not fall into these categories. We suggest further heuristics to judge risk that can augment ongoing efforts to bolster bioscience governance (e.g., the National Academy of Science's interim framework on synthetic biology; National Academies of Sciences, 2017b).

- For a piece of information to be at a high level of risk, that information should be able to provide a significant capacity to cause harm over what is already widely available.
- Many potential risks require several pieces of information to realize. In such cases, information that comprises a “piece of the puzzle” is less hazardous, the greater the number of other pieces that remain unknown. The hardest pieces to discover comprise the greatest hazard, as they reduce the difficulty of a bad actor “solving the puzzle” the most. The hardest piece might not be an experimental result, but an innovation with broad applications, or tacit knowledge for performing a given technique.
- Information can also act as a substitute for other resources in dangerous projects. This suggests that insofar as malicious actors are limited by other factors, they may gain larger benefits from information that can substitute: such information may hence be particularly hazardous.
- Information can become more or less hazardous over time, for example, if the risk relies upon a biotechnological technique that is soon to get dramatically cheaper. Therefore, consider not only hazard at the present moment, but also the forecast hazard when taking account of likely future trends (e.g., the capacity for *de novo* synthesis of organisms of concern relies on their genomes being publicly available).
- Uncertainty about degree of hazard is usually a cause for caution rather than reassurance, given the larger consequences if one has mistakenly underestimated rather than overestimated the degree of hazard (Ord, Hillerbrand, & Sandberg, 2010). A corollary is that information that better clarifies the hazard is very valuable.

### 4.2. Describe How (and How Much) the Information Helps Good and Bad Actors

In all of these case studies, the potentially hazardous information also had potential benefits. The degree of hazard can be clarified by considering what scenarios are of greatest concern.

For Botulinum toxin H, the “worst‐case scenario” appears more a new weapon of terror than a global catastrophe: the challenges observed in prior biological weapons programs of distributing even highly lethal toxins make them infeasible means of massive casualties for state actors, and putative nonstate actors would need considerable sophistication to translate the sequence into a weapon (although the difficulty is steadily falling). Weighed across the scales of faster risk assessment and response, the balance may weigh in favor of (at least limited) disclosure.

For mousepox, the “worst‐case scenario”—a deliberate release of modified, vaccine‐resistant smallpox—threatens a catastrophe that threatens human civilization, and the insight provided by the mousepox work a much larger piece of the puzzle for motivated bad actors. The hazard weighs heavier, and so the rationales for disclosure (such as to “raise the alarm”) may have to clear a higher bar.

### 4.3. Disclose Hazardous Information in the Way That Maximally Disadvantages Plausible Bad Actors

A security objective when disclosing hazardous biological information is providing the greatest differential benefit to good actors over bad actors, so disadvantaging the latter the most.

The Botulinum toxin H case suggests that there are occasions where secrecy is not safer: wider disclosure of information may have brought more benefits in terms of accelerated risk assessment and (if necessary) development of countermeasures than risks of empowering bad actors.

Some cases pose a dilemma where greater or lesser disclosure empowers different bad actors. The gain‐of‐function experiments are one example, if we consider one of the “bad actors” nature itself: greater disclosure aids deliberate misusers, while less disclosure “aids” naturally occurring H5N1 by sequestering knowledge that aids its monitoring and control.

Intermediate or nuanced strategies may provide the greatest differential benefit. Verification of who is accessing particular information is one possibility. “Security by obscurity” is another, ranging from deliberately refraining from highlighting or publicizing means of misuse that could be inferred from a given discovery, to more passive decisions around using jargon easily interpretable by professional scientists, to exploiting the fact that “tacit knowledge” of the know‐how of bioscience work is seldom communicated in the scientific literature, and may compose an important barrier to biotechnological misuse (Ougrham‐Gormley, 2014). (Each of these have their own costs, and may not be resilient in the face of democratizing science and a more accessible scientific literature.) Finding further options intermediate between complete transparency and opacity should be prioritized.

## 5. CONCLUSION

Hazard of information misuse is not the only consideration that informs whether a given piece of biological research should be performed, or its results published. Yet, it is a consideration, and one growing in importance given the increasing power and dissemination of biotechnology. The information hazard case studies we describe are also, strikingly, among the highest‐profile examples of dual‐use research of concern, yet the biosecurity emphasis remains on physical material instead of information. We need better tools and approaches to address biological information hazards.

Decision making in light of this consideration is best done early if possible, and embedded into good research governance. The cases above, and others besides (the demonstration of *de novo* horsepox synthesis perhaps the most recent example) (Kupferschmidt, 2018), suggest that the status quo sometimes fails. The costs of future failures increase in step with the march of biotechnological progress. We hope this article starts to point to a better way.
